# Validation of the quantitative point-of-care CareStart biosensor for assessment of G6PD activity in venous blood

**DOI:** 10.1371/journal.pone.0196716

**Published:** 2018-05-08

**Authors:** Germana Bancone, Gornpan Gornsawun, Cindy S. Chu, Pen Porn, Sampa Pal, Pooja Bansil, Gonzalo J. Domingo, Francois Nosten

**Affiliations:** 1 Shoklo Malaria Research Unit, Mahidol–Oxford Tropical Medicine Research Unit, Faculty of Tropical Medicine, Mahidol University, Mae Sot, Thailand; 2 Centre for Tropical Medicine and Global Health, Nuffield Department of Medicine, University of Oxford, Oxford, United Kingdom; 3 Diagnostics Program, PATH, Seattle, Washington, United States of America; Academic Medical Centre, NETHERLANDS

## Abstract

**Introduction:**

Glucose-6-phosphate dehydrogenase (G6PD) deficiency is the most common enzymopathy in the human population affecting an estimated 8% of the world population, especially those living in areas of past and present malaria endemicity. Decreased G6PD enzymatic activity is associated with drug-induced hemolysis and increased risk of severe neonatal hyperbilirubinemia leading to brain damage. The G6PD gene is on the X chromosome therefore mutations cause enzymatic deficiency in hemizygote males and homozygote females while the majority of heterozygous females have an intermediate activity (between 30–80% of normal) with a large distribution into the range of deficiency and normality. Current G6PD qualitative tests are unable to diagnose G6PD intermediate activities which could hinder wide use of 8-aminoquinolines for *Plasmodium vivax* elimination. The aim of the study was to assess the diagnostic performances of the new Carestart G6PD quantitative biosensor.

**Methods:**

A total of 150 samples of venous blood with G6PD deficient, intermediate and normal phenotypes were collected among healthy volunteers living along the north-western Thailand-Myanmar border. Samples were analyzed by complete blood count, by gold standard spectrophotometric assay using Trinity kits and by the latest model of Carestart G6PD biosensor which analyzes both G6PD and hemoglobin.

**Results:**

Bland-Altman comparison of the CareStart normalized G6PD values to that of the gold standard assay showed a strong bias in values resulting in poor area under-the-curve values for both 30% and 80% thresholds. Performing a receiver operator curve identified threshold values for the CareStart product equivalent to the 30% and 80% gold standard values with good sensitivity and specificity values, 100% and 92% (for 30% G6PD activity) and 92% and 94% (for 80% activity) respectively.

**Conclusion:**

The Carestart G6PD biosensor represents a significant improvement for quantitative diagnosis of G6PD deficiency over previous versions. Further improvements and validation studies are required to assess its utility for informing radical cure decisions in malaria endemic settings.

## Introduction

8-aminoquinolines (such as primaquine and tafenoquine) are the only drugs able to kill the dormant liver stages (hypnozoites) of *Plasmodium vivax*. Hypnozoites can be activated and cause new infections (relapses) without the need of new mosquito bites. In vivax endemic areas of South-East Asia, relapses are considered to cause up to 80% of the overall malaria episodes contributing enormously to malaria morbidity and mortality [1–4]. Wide use of 8-aminoquinolines in malarious areas is hindered by the associated hemolytic risk in glucose-6-phosphate dehydrogenase (G6PD) deficient patients [5–7] and by the lack of suitable field testing. G6PD deficiency is the most common enzymopathy in the human population with an estimated 8% prevalence worldwide and up to >30% prevalence in populations living in areas of past and present malaria transmission [8]. G6PD is an essential enzyme for maintaining the redox equilibrium in all cells and especially in red blood cells where a nucleus is lacking. G6PD deficiency can be caused by several mutations in the G6PD gene which cause protein instability and decreased enzymatic activity [9, 10]. Since the G6PD gene is on the X chromosome, a strict correlation between phenotype and genotype only exists for the hemizygous and homozygous genotypes. In heterozygous females, due to early random inactivation of the X-chromosome, only one of the genes is expressed in each cell. In the blood, a variable proportion of G6PD normal and G6PD deficient red blood cells can be found giving rise to a range of phenotypes [11]. The majority of heterozygous females have an intermediate activity between 30% and 80% of normal; in the case of one of the major South-East Asian variants, Mahidol, around 7% of heterozygous women have an activity <30% of normal and 18% have an activity >80% of normal [12]. Current G6PD qualitative rapid tests only diagnose deficiency under the 30% threshold [13–16] and women with intermediate activity are diagnosed as G6PD normal by these tests. At the Thai-Myanmar border and in most of the South-East Asian countries where *P*. *vivax* malaria is still endemic, the prevalence of G6PD deficiency among males ranges from 10 to 20% [17] with a correspondent percentage of heterozygous women ranging from 20 to 30%. As a consequence, primaquine is currently either administered in many women with a relatively high risk of hemolysis (in countries where treatment is dependent on RDT testing) [5], or it is not given at all due to safety concerns [18] in a large proportion of the population. At the same time, with the currently available qualitative tests, Tafenoquine cannot be administered safely to any woman because it should only be prescribed in patients with >70% G6PD activity [7].

In populations with high prevalence of G6PD deficiency, quantitative G6PD testing can have a broader use; identifying G6PD deficient persons who have common clinical conditions requiring antibiotic treatment so that oxidative drugs such as nitrofurantoin, nalidixic acid, ciprofloxacin and rasburicase can be avoided [19, 20]. Furthermore, G6PD deficiency is associated with increased susceptibility to neonatal hyperbilirubinemia and bilirubin-induced brain damage [21, 22]; testing for G6PD deficiency on umbilical cord blood or very early in the neonatal period using qualitative rapid tests has been shown to be unreliable [23] because of reticulocytosis [24]. Thus, quantitative G6PD tests in these populations can play a role in diagnosing G6PD deficiency more accurately in neonates.

Laboratory based quantitative testing for G6PD is carried out using a relatively expensive spectrophotometric assay [25] that requires a well equipped laboratory, skilled technicians and reliable source of material. The majority of populations with high prevalence of G6PD deficiency live in tropical countries [8] where this test is available only in specialized hospitals or not available at all. Quantitative G6PD point-of-care (POC) tests are an important tool for wider and safer use of 8-aminoquinolines which are needed for malaria elimination, for safe prescription of oxidative drugs in common clinical conditions, and for a more accurate diagnosis of G6PD deficiency at birth to improve the diagnosis and management of neonatal hyperbilirubinemia. These devices have been developed in recent years [26]. The aim of the study was to assess in a laboratory setting the diagnostic performances of the latest Carestart G6PD quantitative biosensor against the gold standard spectrophotometric assay in blood samples collected from healthy volunteers.

## Materials and methods

### Study site and population

The study was conducted along the Thai-Myanmar border (Tak Province) at Shoklo Malaria Research Unit (SMRU); SMRU serves a migrant population composed of Burman and Karen ethnic groups through two clinical sites located north and south to Mae Sot. Volunteers aged >18 years were recruited at the clinics and, after giving written informed consent, their blood was collected; samples were refrigerated and shipped in cool boxes to the central hematology laboratory in Mae Sot where analyses were performed.

### Study design

A targeted enrollment strategy was used as in Bancone *et al*. 2015. In brief, individuals with known G6PD status were invited to join the study in order to achieve a convenience sample of approximately 50 G6PD deficient volunteers (male and female), approximately 50 G6PD heterozygous female volunteers, and 50 G6PD-normal volunteers. Targeted enrollment allowed for comparing the new device against the gold standard spectrophotometric assay across the entire range of enzymatic activities without the need to screen a large number of subjects.

### Blood sampling

Five hundred microliters of venous blood were drawn from the arm, transferred to a K2 EDTA tube (BD Vacutainer^™^, USA) and inverted 10 times; samples were kept at 4°C until analysis.

### Laboratory procedures

All laboratory procedures were conducted in air-conditioned room.

A complete blood count was performed on samples within 8 hours from blood collection. The G6PD quantitative spectrophotometric assay and the quantitative POC CareStart assay were performed the following day (within 30 hours from blood collection).

The CBC was performed using a CeltacF MEK-8222K hematology analyzer (Nihon Kohden, Japan). The CBC included white blood cells (WBC) total and differential count, red blood cells number (RBC), red blood cells size (MCV), hemoglobin content (MCH and MCHC), total hemoglobin concentration (HGB), hematocrit (HCT) and platelets count (PCT). Quality controls were run every day before analysis of samples.

The G6PD spectrophotometric assay (Trinity Biotech, Ireland) was performed in duplicate using 10μL of whole blood per replicate; instructions from supplier were followed for reagents preparation. An UV-1800 SHIMADZU (Japan) spectrophotometer with an electronically controlled temperature compartment was used to detect the absorbance at 340 nm during 10 minutes at 30°C. G6PD activity was calculated as IU/gHb using the results of the complete blood count on the same blood.

**Fig 1:**
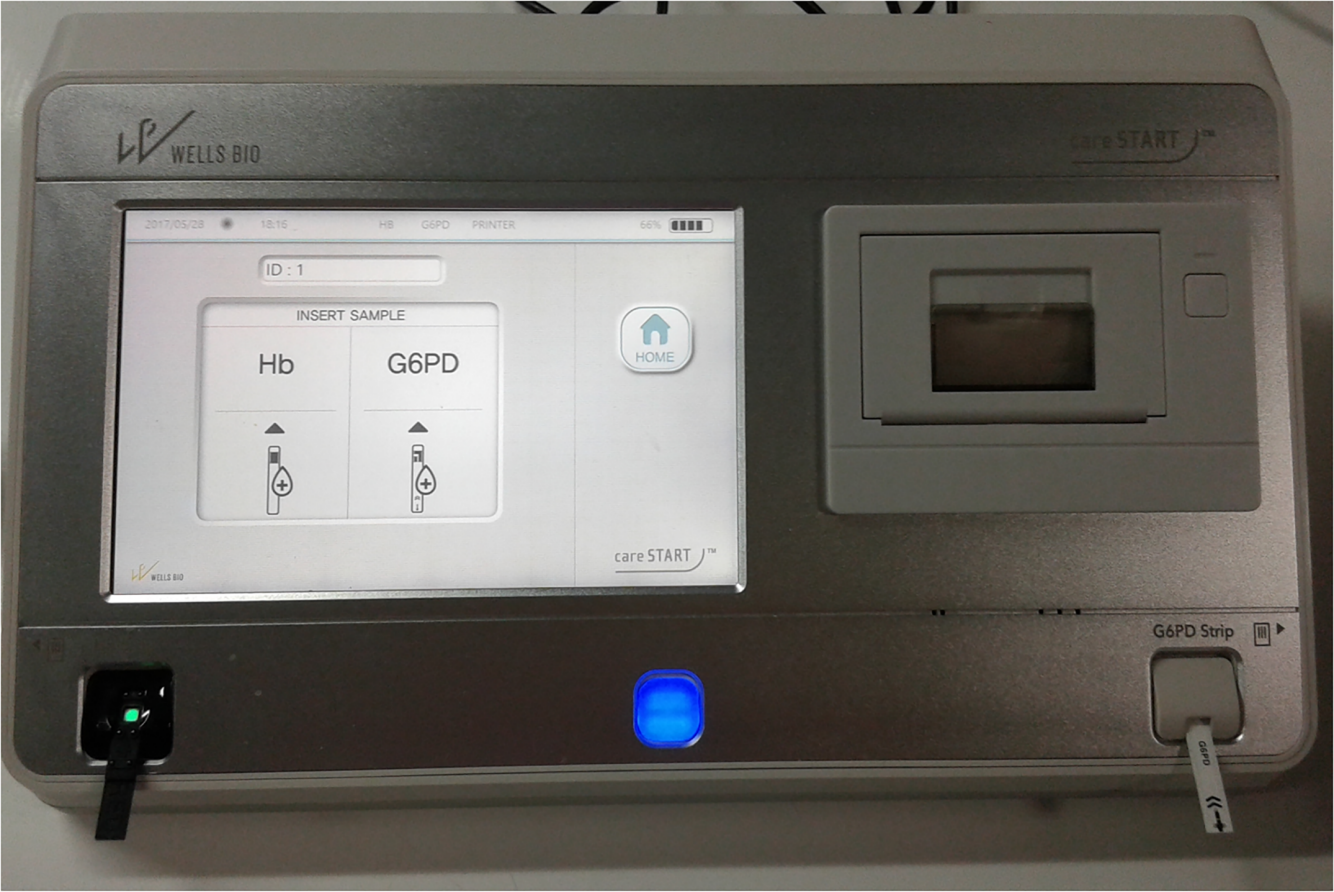
CareStart G6PD biosensor. The device consolidates a hemoglobin measurement device (on the left) with a G6PD activity measurement device (on the right). Two disposables and samples are therefore required, one of each analyte measurement. The device then calculates the G6PD activity normalized for hemoglobin concentration based on the two measurements.

The latest CareStart biosensor model (WellsBio, USA), which analyses both G6PD and Hb, was used (Fig 1). The tests were performed according to the manufacturer’s instructions; both Hb and G6PD activity controls were run each day before samples. Seven μL of blood were transferred to the Hb strip using a professional pipette while 7 μL of blood were spotted on parafilm prior to be transferred to the G6PD strip. After 5 minutes the CareStart biosensor reported both the concentration of Hb in g/dL and the activity of G6PD as U/gHb. Each sample was run in duplicate.

Notes from the laboratory operators who used the CareStart Biosensor during the study were collected to describe some aspects of usability. Prices for the biosensor and for the associated strips at the time of manuscript submission were not available, therefore no cost analysis was performed during the study.

### Statistical methods

The G6PD quantitative spectrophotometric assay was used as the reference assay. Population median was calculated on normal males as already reported before [14]. Correlation between the two replicates of CareStart biosensor was analyzed by linear regression. Area under the curve (AUC) of the receiving operator characteristic (ROC) curve [27] was calculated at different activity thresholds to analyze clinical performances of the Biosensor. Youden’s Index for CareStart biosensor was calculated [27]. All statistical analyses were conducted in Stata 13.0 (Statacorp, College Station, TX).

### Ethical approval

Ethical approval was obtained by the Ethics Committee of the Faculty of Tropical Medicine, Mahidol University (FTMEC MO/15/259) and by Oxford Tropical Research Ethics Committee (OXTREC 563–15).

## Results

### Study population

A total of 150 subjects were recruited in the study, 42 males and 108 females. Mean age (±SD) was 37.1 (12.4) in males and 31.0(9.3) in females. The majority of participants were of Sgaw Karen (89; 59.4%) and Poe Karen (28; 18.7%) ethnicity; the remaining were Burman (22; 14.7%) and individual of other ethnicities (11; 7.3%). Participants were healthy; none of them had malaria, 5 (3.3%) had medical complaints and 15 women (13.9%) were pregnant. Blood characteristics for the study sample are reported in Table 1.

**Table 1:** Hematologic characteristics of study population by gender.

Sex		WBC (10^3^/μL)	RBC (10^6^/μL)	HCT (%)	HGB (g/dL)	PLT (10^3^/μL)
Males (N = 42)	Mean	6.5	4.92	43.6	14.1	257
	SD	1.5	0.45	3.3	1.2	70
Females (N = 108)	Mean	7.6	4.49	39.3	12.8	294
	SD	2.2	0.52	3.4	1.2	67
Total (N = 150)	Mean	7.3	4.61	40.5	13.2	284
	SD	2.1	0.54	3.9	1.3	70

### Use of the CareStart G6PD biosensor

The device was run on power supply in air-conditioned laboratory conditions; testing strips were kept at room temperature in the same laboratory. Blood samples were handled by laboratory technicians and locally trained laboratory staff and blood transfer was carried out using precision pipettes; manual instructions were followed without a previous training. The machine carried out simultaneous analysis of Hb levels and G6PD activity (Fig 1) using two different methods for blood transfer: for the Hb the blood needed to be transferred through a pipette on the Hb strip membrane while for the G6PD the sample could be transferred directly from the finger prick. When transferring the samples from a collection tube, as in our validation study, the blood needed to be spotted first on parafilm and then transferred to the G6PD strip. Since the direct transfer from finger prick would not allow for volume measurement and the instruction manual did not specify the required volume, we tested the transfer of volumes of blood between 4μl and 9μl. Results with blood volumes lower than <5μl were not correct but the machine did not show any alarm for insufficient sample volume.

Results were displayed within five minutes and could be printed or stored in the machine (up to 5,000 results including date, time and measured temperature). No error messages were encountered during the whole validation study and operators had not trouble running the device.

### Clinical performances of the CareStart G6PD biosensor

Correlation in G6PD activity and Hb levels between the two replicates of the same sample using the CareStart biosensor were R^2^ = 0.83 and R^2^ = 0.94, therefore the average for each sample could be used to compare the performances of the new device against the gold standard.

**Fig 2:**
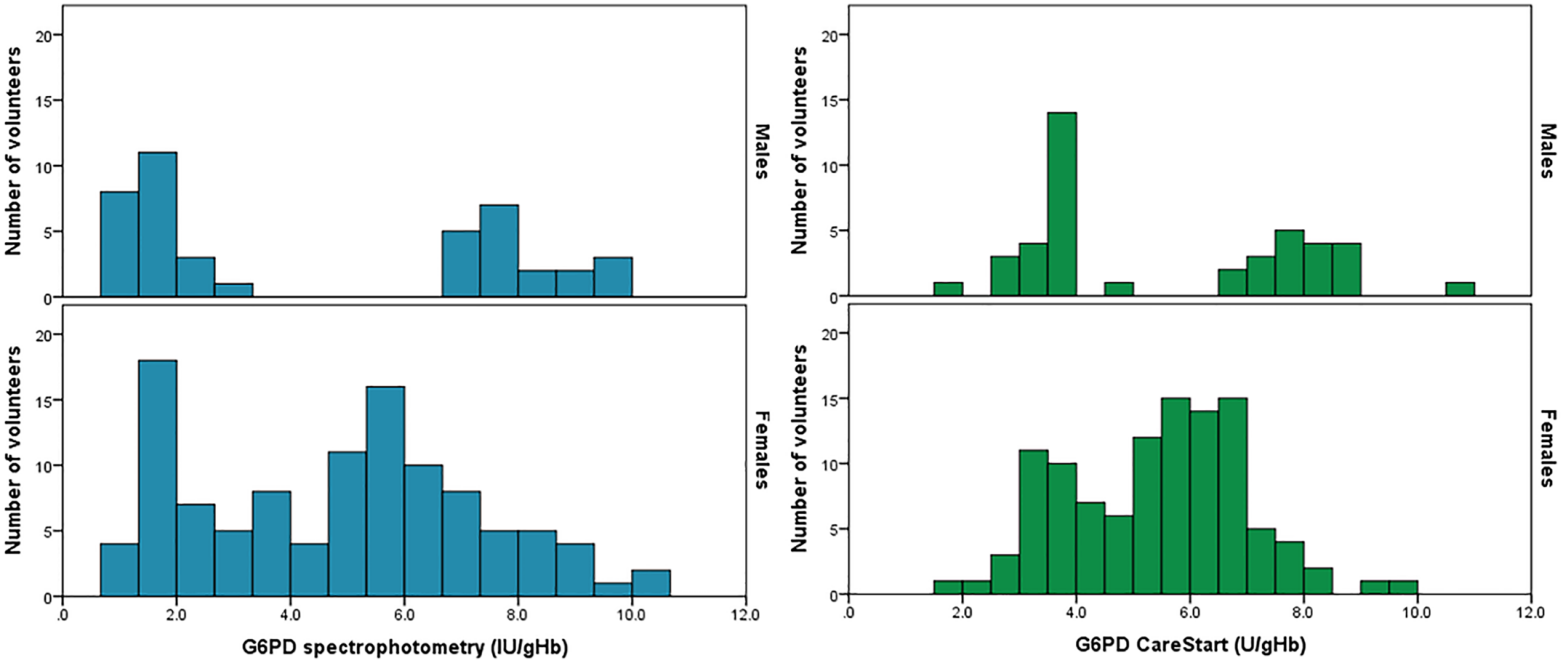
Distribution of G6PD activity by gender and diagnostic device. The left pane shows the results of the gold standard spectrophotometric assay, the right pane shows the results of the CareStart biosensor.

Furthermore, hemoglobin concentration assessed by the biosensor was in good agreement with the results from the complete blood count analysis (R^2^ = 0.88) so the enzymatic activity expressed as IU/gHb was used for comparisons between the two techniques. Distributions of G6PD activity assessed by the gold standard and by the CareStart biosensor are reported in Fig 2 by gender; both tests showed a clear bimodal distribution for samples from male volunteers and a continuous distribution for samples from female volunteers; the whole population had a similar range of activities by both tests, minimum activities assessed by the gold standard and by the CareStart biosensor were 1.85 IU/gHb and 0.84 IU/gHb respectively and maximum activities were 10.50 IU/gHb and 10.23 IU/gHb respectively.

Median activity by spectrophotometry in normal males was 7.91IU/gHb; based on this value, the 30% threshold corresponded to 2.37IU/gHb and 80% threshold corresponded to 6.33IU/gHb. Median activity assessed by the CareStart biosensor in normal males was 7.85IU/gHb; based on this value, the 30% threshold corresponded to 2.36IU/gHb and 80% threshold corresponded to 6.28IU/gHb.

**Fig 3:**
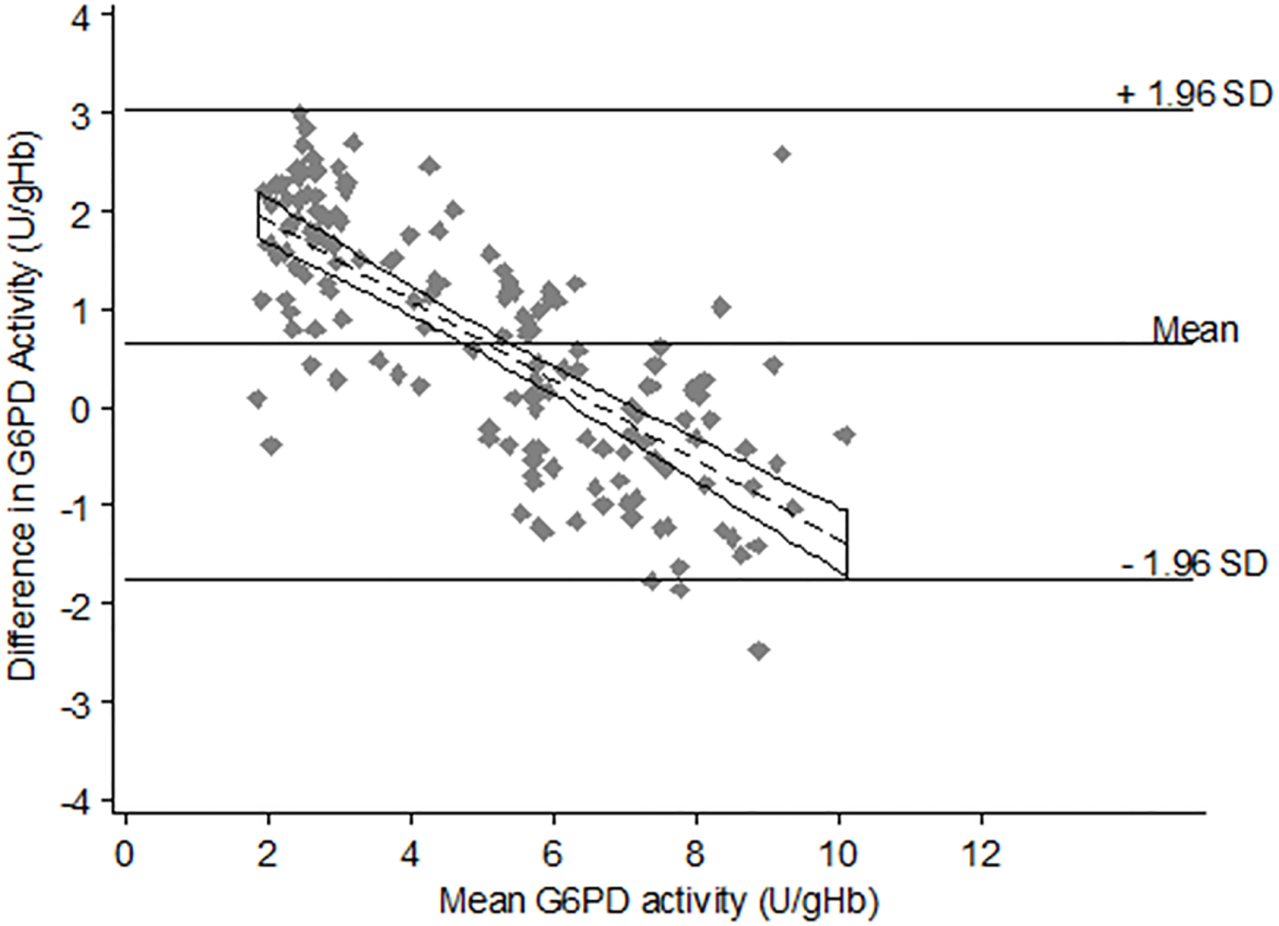
Bland-Altman plot for G6PD activity with regression line.

The Bland-Altman plot for comparison of G6PD activity by the CareStart biosensor against the gold standard (Fig 3) showed a large confidence interval with the majority of samples falling within the 1.96SD from the mean difference. Data were not scattered around the mean difference particularly in samples with low G6PD activity (left side of the plot) where the data were all above the mean showing that the CareStart biosensor overestimated activity as compared to the spectrophotometry.

Assuming equivalency in percent thresholds for the spectrophotometric reference assay and the CareStart product and analyzing the AUC for the ROC curve at different activity thresholds (Table 2), CareStart biosensor performed poorly in detecting deficient subjects at the 30% spectrophotometric activity threshold (<2.37IU/gHb) with an AUC of 0.52. Using Youden’s Index (Table 3), the optimal activity threshold of CareStart for diagnosis of samples with <30% spectrophotometric activity was estimated to be 4.55IU/gHb; at this threshold the test had 100% sensitivity and 92% specificity. The optimal threshold of CareStart for diagnosis of samples with <80% activity was 6.10 IU/gHb, with a sensitivity of 92% and a specificity of 94%.

**Table 2:** Area under the curve for ROC analysis at different activity thresholds for the reference test and CareStart biosensor.

Threshold*	Gold Standard G6PD value	CareStart G6PD value	All samples	Only females
	IU/g Hb	IU/g Hb	AUC (95%CI)	AUC (95%CI)
30%	2.37	2.36	0.52 (0.49–0.55)	0.52 (0.48–0.55)
40%	3.16	3.14	0.64 (0.58–0.70)	0.68 (0.60–0.77)
70%	5.54	5.50	0.85 (0.79–0.90)	0.79 (0.71–0.86)
80%	6.33	6.28	0.91 (0.86–0.96)	0.86 (0.78–0.94)

*Assuming equivalency in G6PD activity thresholds in terms of % of normal activity

**Table 3:** Threshold normalized G6PD activity values on the CareStart biosensor optimized to maximize sensitivity and specificity using Youden’s Index.

**30% Threshold on Gold Standard assay (2.37 IU/g Hb)**	
Optimal Threshold G6PD activity (IU/g Hb)	4.55
Sensitivity at Threshold (95%CI)	1.00 (0.89–1.00)
Specificity at Threshold (95%CI)	0.92 (0.86–0.97)
Youden’s Index	0.92
**40% Threshold on Gold Standard assay (3.16 IU/g Hb)**	
Optimal Threshold G6PD activity (IU/g Hb)	4.55
Sensitivity at Threshold (95%CI)	0.96 (0.83–1.00)
Specificity at Threshold (95%CI)	0.97 (0.91–0.99)
Youden’s Index	0.93
**70% Threshold on Gold Standard assay (5.54 IU/g Hb)**	
Optimal Threshold G6PD activity (IU/g Hb)	5.15
Sensitivity at Threshold (95%CI)	0.79 (0.67–0.86)
Specificity at Threshold (95%CI)	0.98 (0.92–1.00)
Youden’s Index	0.77
**80% Threshold on Gold Standard assay (6.33 IU/g Hb)**	
Optimal Threshold G6PD activity (IU/g Hb)	6.1
Sensitivity at Threshold (95%CI)	0.92 (0.85–0.97)
Specificity at Threshold (95%CI)	0.94 (0.83–0.99)
Youden’s Index	0.86

## Discussion

Use of high dose primaquine and tafenoquine for radical cure of *P*. *vivax* malaria is safe in G6PD normal patients. While G6PD deficiency can be diagnosed by qualitative tests in both males and females, female patients with intermediate G6PD activity who are at risk of hemolysis [5] cannot be diagnosed reliably by current qualitative tests. New devices for the quantitative assessment of G6PD activity in *P*. *vivax* patients are urgently needed for elimination of the disease. Furthermore, quantitative POC instruments might give a faster assessment of G6PD status and facilitate the safe prescription of oxidative antibiotic treatment in remote clinical settings. A more reliable diagnosis of G6PD deficiency at birth [28] can improve the diagnosis and management of neonatal hyperbilirubinemia [22]. Ideally, the absolute results from a quantitative G6PD POC test can be compared with the gold standard reference test. More importantly, there should be high sensitivity and specificity at defined threshold points for activity calculated from the relative adjusted male median [29, 30]. Moreover, the test should be able to provide simultaneous results for G6PD activity and hemoglobin levels for quantification of enzyme in terms of IU/gHb.

The CareStart biosensor was recently developed; while previous models would only allow for quantification of G6PD activity [26, 31], a new relatively compact machine was developed recently and was tested in this study. This model was designed to perform both G6PD enzyme and Hb analyses in the same device (Fig 1) from blood samples collected by finger prick or venipuncture. The biosensor was easily operated by laboratory technicians and locally trained laboratory staff following the instruction manual. Analysis of Hb could be performed as part of the G6PD testing or as a stand-alone test, showing a good flexibility for use in clinical settings. A few improvements in the design of the blood transfer would be beneficial, including using the same blood transfer method and the possibility to operate the device with smaller sample volumes (currently a total blood volume of at least 14μl is required).

When compared to the gold standard spectrophotometric assay, the results by the CareStart biosensor were very similar in G6PD normal males but much higher in deficient volunteers, both males and females. Nevertheless, using a higher activity threshold (in this case 4.55IU/gHb) maximized the sensitivity and specificity of the test for diagnosis of deficient samples with <30% activity by spectrophotometry. Using this threshold gave a good diagnostic power in both male and female population to discriminate deficient subjects from the others.

For the female population only, where discrimination of intermediate and normal activities is paramount for correct deployment of primaquine and especially tafenoquine, the sensitivity and specificity were acceptable (90% and 92.5% respectively) when a threshold of 6.10IU/gHb for 80% activity was used. Thus, the device showed improved performances compared to previous versions but the ideal threshold points for discriminating samples with activity between 30% to 80% normal were alarmingly narrow, with a range of only 1.55IU/gHb (4.55–6.10).

Since the quantification of Hb levels was good, the observed differences between the enzymatic activities assessed by the Biosensor as compared to the spectrophotometric assay were most probably dependent on the detection of changes in absorbance or in the algorithm used for calculation of activity as IU/gHb.

It is important that any quantitative G6PD Biosensor machine is extensively validated before use in clinical trials to avoid non-reproducible data [32] or, worse, misdiagnosis in G6PD deficient patients who are at risk of hemolysis from oxidative treatments. This study was performed in a laboratory setting with highly regulated environmental temperature and humidiy settings. Testing the device over multiple environmental conditions more reflective of the intended use in hot tropical climates will be critical to further validate its performance and robustness. Specific validation studies will be needed to assess the performances of the machine in diagnosing G6PD deficiency in newborns using cord blood samples.

## Conclusions

The current data shows that the performances of the CareStart biosensor have improved significantly over previous evaluations. Careful considerations need to be made to set threshold G6PD values that inform case management when oxidative drugs will be prescribed. Further validations studies are required to replicate the performance of the test under multiple environmental conditions, different specimens and with different end-users. Considering the slightly more complex steps needed for using a biosensor, only a device with comparable performances to the gold standard spectrophotometry (i.e. superior performances as compared to qualitative test) would be a suitable candidate for replacing currently available G6PD rapid diagnostic tests.

## Supporting information

S1 Table Study dataset. (XLSX)
